# Relationship between the Soluble F11 Receptor and Annexin A5 in African Americans Patients with Type-2 Diabetes Mellitus

**DOI:** 10.3390/biomedicines10081818

**Published:** 2022-07-28

**Authors:** Ajibola Adedayo, Ayobami Eluwole, Fasika Tedla, Arye Kremer, Muhammad Khan, Nicole Mastrogiovanni, Carl Rosenberg, Paul Dreizen, John La Rosa, Louis Salciccioli, Mohamed Boutjdir, Mary Ann Banerji, Clinton Brown, Jason Lazar, Moro Salifu, Ahmed Bakillah

**Affiliations:** 1 Department of Medicine, Downstate Medical Center, State University of New York, 450 Clarkson Ave., Brooklyn, New York, NY 11203, USA; ajibola.adedayo@downstate.edu (A.A.); ayobami.eluwole@downstate.edu (A.E.); fasika.tedla@downstate.edu (F.T.); arye.kremer@downstate.edu (A.K.); muhammad.khan@downstate.edu (M.K.); nicole.mastrogiovanni@downstate.edu (N.M.); paul.dreizen@downstate.edu (P.D.); john.larosa@downstate.edu (J.L.R.); louis.salciccioli@downstate.edu (L.S.); mohamed.boutjdir@downstate.edu (M.B.); maryann.banerji@downstate.edu (M.A.B.); clinton.brown@downstate.edu (C.B.); jason.lazar@downstate.edu (J.L.); moro.salifu@downstate.edu (M.S.); 2 Department of Epidemiology and Biostatistics, School of Public Health, SUNY Downstate Health Sciences University, 450 Clarkson Ave., Brooklyn, New York, NY 11203, USA; carl.rosenberg@downstate.edu; 3 King Abdullah International Medical Research Center (KAIMRC), King Saud bin Abdulaziz University for Health Sciences (KSAU-HS), Ministry of National Guard Health Affairs, Al Ahsa 31982, Saudi Arabia

**Keywords:** annexins, biomarkers, cardiovascular disease, diabetes, endothelium function, F11R, vascular complications, vascular reactivity index, artery stiffness

## Abstract

Type 2 diabetes mellitus (T2DM) is characterized by endothelial dysfunction, increased thrombogenicity, and inflammation. The soluble human F11 receptor (sF11R) and annexin A5 (ANXA5) play crucial roles in inflammatory thrombosis and atherosclerosis. We examined the relationship between circulating sF11R and ANXA5 and their impact on endothelial function. The study included 125 patients with T2DM. Plasma levels of sF11R and ANXA5 were quantified by ELISA. Microvascular function was assessed using the vascular reactivity index (VRI). Large artery stiffness was assessed by carotid-femoral pulse wave velocity (PWV). Carotid intima-media thickness (CIMT) was assessed by B-mode ultrasound imaging. The mean age of patients in the study was 59.7 ± 7.8 years, 78% had hypertension, 76% had dyslipidemia, and 12% had CKD. sF11R correlated positively with ANXA5 levels (β = 0.250, *p* = 0.005), and correlated inversely with VRI and total nitic oxide (NO), (β = −0.201, *p* = 0.024; β = −0.357, *p* = 0.0001, respectively). Multivariate regression analysis revealed that sF11R was independently associated with ANXA5 in the total population and in patients with HbA1c > 6.5% (β = 0.366, *p* = 0.007; β = 0.425, *p* = 0.0001, respectively). sF11R and ANXA5 were not associated with vascular outcome, suggesting that they may not be reliable markers of vascular dysfunction in diabetes. The clinical significance of sF11R/ANXA5 association in diabetes warrants further investigation in a larger population.

## 1. Introduction

Patients with T2DM have a markedly increased risk of developing cardiovascular disease (CVD). Beside blood glucose level abnormalities, microvascular and macrovascular complications associated with T2DM are mainly triggered by metabolic changes that affect the vascular wall, including insulin resistance, endothelial dysfunction, oxidative stress, low grade inflammation, and platelet hyperactivity [1,2]. Recent studies have shown that patients with T2DM have increased thrombogenicity characterized by the activation of coagulation factors, platelet hyperactivity, and hypofibrinolysis [3,4,5]. Remarkable racial differences in intrinsic thrombogenic properties and response to anti-thrombotic agents have been reported among various ethnic populations, with African Americans having the most thrombogenic state and higher risk for atherothrombotic events [6,7,8].

Hyperglycaemia leads to impairment of NO production [9]. Moreover, endothelial dysfunction is associated with impaired NO availability [10]. Several studies reported altered NO levels in T2DM, but the data were very controversial. Some studies reported increased NO levels in diabetic patients, whereas others reported the opposite [9,11,12]. NO regulates both vascular tone and platelet function [13]. Coronary atherothrombotic disease has been associated with abnormal NO release or decrease in NO bioavailability. NO is released by the endothelium, preventing platelet adhesion to the vessel wall. When released by platelets, NO inhibits the further recruitment of platelets to the growing thrombus [14]. The relationship between circulating NO and plasma sF11R or ANXA5 levels is not well established.

The F11 receptor (F11R; aca JAM-A; JAM-1) is a cell adhesion protein expressed on the cell membrane of circulating platelets and present within tight junctions of endothelial cells [15,16]. F11R is involved in the adhesion of platelets to cytokine-inflamed endothelial cells, suggesting a role in the initiation of atherosclerotic plaque formation [17]. Studies have demonstrated significant elevation of circulating sF11R in hypertensive and hemodialysis patients [18,19]. ANXA5, a member of annexin superfamily, is a protein known for its antithrombotic properties, which are mediated mainly by the mechanical shielding of phospholipids, particularly phosphatidylserine, which result in reducing their availability for coagulation reactions [20,21]. ANXA5 may play a role in CVD, as it was found to be abundant in late-stage atherosclerotic lesions [21]. It is also involved in the metastasis, invasion, and development of cancer cells [22], playing an important role in the process of cell plasma membrane repair [23]. Antibodies against ANXA5 have been shown to interfere with ANXA5 functions, leading to thrombotic complications during diabetes [24].

sF11R and ANXA5 are both involved in atherogenesis; however, limited data exist on the relationship between sF11R and ANXA5 levels and their impact on vascular function and atherosclerotic burden in T2DM patients. In this study, we hypothesized that changes in circulating sF11R and ANXA5 could influence indices of endothelial dysfunction and subclinical atherosclerosis in patients with poorly-controlled T2DM.

## 2. Materials and Methods

### 2.1. Study Population and Protocol

A total of 125 African Americans patients with T2DM were recruited from the State University of New York Downstate Health Sciences University/Kings County Clinics between September 2016 and July 2017. The study protocol was approved by the Institutional Review Board of the State University of New York Downstate Health Sciences University (IRB protocol# 907067), and written informed consent was obtained from each participant. Patients were excluded from the study if, at baseline, patients met one or more of the following criteria: patients were receiving chronic renal replacement therapy (hemodialysis, peritoneal dialysis, or transplantation), had a history of active malignancy (except those with basal cell carcinoma) within the last five years (prostatic cancer within the last two years), systemic lupus erythematosus and other autoimmune diseases that may affect kidney function, history of type 1 diabetes mellitus, acute infection or fever, pregnancy, chronic viral hepatitis or HIV infection, current unstable cardiac disease, history of hypercoagulable disorder, history of blood clots in arms, weak pulses in arms indicating low brachial artery flow, or history of vasculitis. Standard methods and definitions were adopted: Diabetes—subjects with history of T2D on medication, or HbA1c ≥ 6.5%, or fasting glucose ≥126 mg/dL (≥7 mmol/L). Dyslipidemia—subjects with history of dyslipidemia on medication, or fasting lipid profile with total cholesterol >200 mg/dL, or LDL >70 mg/dL. Hypertension—subjects with systolic blood pressure ≥140 mmHg or diastolic blood pressure ≥90 mmHg and under antihypertensive medication use. CKD—subjects with eGFR < 90 mL/min, using modification of diet in renal disease (MDRD) equation, or proteinuria (≥2+ on urine dipstick).

### 2.2. Measurement of Circulating sF11R, ANXA5 and Total NO

Fasting blood samples were collected in the morning, after a minimum of 12 h fast, into EDTA-containing tubes and centrifuged at 4 °C at 3000 rpm for 10 min to separate the plasma for biochemical tests. All the samples were aliquoted and stored at −80 °C until further analysis. Plasma levels of sF11R, ANXA5, and total NO were measured in duplicate by using commercially available ELISA kits (Aviva Systems Biology Corp, San Diego, CA, USA, Catalog# OKCD07655; Assaypro LLC, St. Charles, MO, USA, Catalog# EA3601-1; and My BioSource, Inc., San Diego, CA, USA, Catalog #MBS732723, respectively).

### 2.3. Quantification of Vascular Changes

Methods for noninvasive assessment of arterial stiffness and endothelial dysfunction have been described elsewhere [25,26,27]. Vascular reactivity (VRI) is an index of blood vessel responsiveness to stimuli that measures microvascular function using differential distal digital thermal response following proximal peripheral vascular occlusion and release (Endothelix Vendys II, Palo Alto, CA, USA). Pulse wave velocity (PWV) is a measurement of arterial stiffness between two major arteries at the carotid-femoral sites. Actual measurement of carotid-femoral PWV (cf-PWV) was performed using the SphygmoCor system (ArtCor, Sydney, Australia). Carotid intima-media thickness (CIMT) was assessed by high-resolution B-mode ultrasound image analysis using an ultrasound machine (Philips Sonos 7500 Cardiac Ultrasound).

### 2.4. Statistical Analysis

Statistical analyses were performed using SPSS software version 24 (IBM Corp., Armonk, NY, USA). Continuous variables with normal distribution were presented as means ± SD, and non-normally distributed variables were reported as medians (IQR). Comparisons between groups were performed by using the Wilcoxon rank sum test. Categorical variables were presented as frequencies and percentages, and comparisons between groups were performed by using Pearson’s chi-square or Fisher’s exact test. Associations between sF11R, ANXA5, and other variables were assessed using the non-parametric Spearman’s correlation test. Multiple linear regression analysis was performed to evaluate the association between sF11R levels and ANXA5, using based models (I-III) for covariates assessment, including factors such as sex, age, weight, hypertension, stroke, smoking, creatinine, total cholesterol, LDLc, HDLc, triglycerides, HbA1c, duration of diabetes, and total NO. All adjusted β-coefficients were accompanied by approximate 95% confidence limits. Two-sided tests with *p*-values < 0.05 were considered statistically significant.

## 3. Results

### 3.1. Clinical and Biochemical Characteristics of the Study Population

The baseline characteristics of the study subjects are shown in Table 1. The patient population was categorized in two groups (HbA1c ≤ 6.5%, N = 27 vs. HbA1c > 6.5%, N = 98). The mean HbA1c levels were 8.06% ± 2.02%; the mean patient age was 59.7 ± 6.8 years (female 63%). A total of 78% had hypertension, 76% had dyslipidemia, and 12% had chronic kidney disease. Among all the parameters, waist circumference, diastolic blood pressure, HbA1c levels, and duration of diabetes were significantly elevated in the poorly-controlled group of participants as opposed to the well-controlled group. The use of medication did not differ significantly between the groups, except that the percentage of patients using calcium channel blockers was significantly higher in the poorly-controlled patients than in the well-controlled patients.

**Figure 1:**
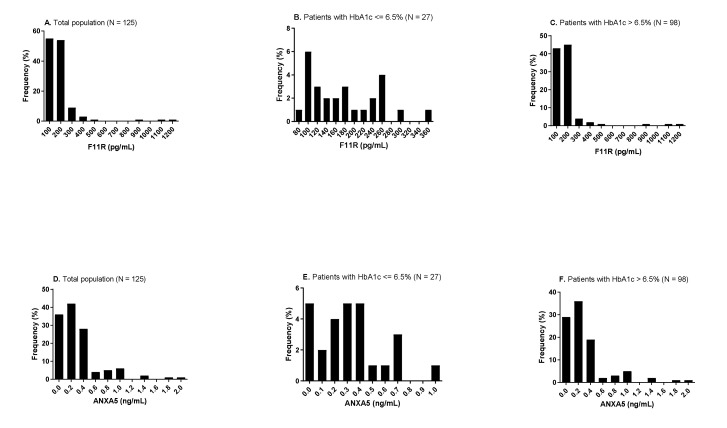
Frequency distribution of plasma sF11R and ANXA5 in the total population and stratified groups based on HbA1c levels. Levels of sF11R in the total population (**A**), and in well-controlled (**B**) poorly-controlled patients (**C**). Levels of ANXA5 in the total population (**D**), and in well-controlled (**E**) poorly-controlled patients (**F**).

Plasma levels of sF11R varied by an almost 20-fold range among the participants in this cohort (56 pg/mL to 1155 pg/mL). The distribution of sF11R levels was right skewed, with a median value of 159 (115.92–199.02) pg/mL in the total population (Figure 1A). ANXA5 varied in a similar right-skewed pattern, with a median value of 0.22 (0.07–0.39) ng/mL in the total population (Figure 1D). Distribution profiles of both sF11R and ANXA5 levels were maintained in poorly-controlled patients, with HbA1c levels > 6.5% (Figure 1C–F), but exhibited the appearance of a bimodal shape in well-controlled patients, with HbA1c levels ≤ 6.5% (Figure 1B–E).

### 3.2. Correlations between Plasma sF11R, ANXA5 and Other Clinical Variables

In the total population, sF11R levels correlated inversely with VRI outcome and total NO levels (r = −0.201, *p* = 0.024 and r = −0.357, *p* = 0.0001, respectively; Table 2) and correlated positively with ANXA5 levels (r = 0.250, *p* = 0.005, Table 2). Similarly, ANXA5 levels correlated negatively with VRI outcome and total NO levels (r = −0.179, *p* = 0.049 and r = −0.351, *p* = 0.0001, respectively; Table 2). In patients with HbA1c ≤ 6.5%, ANXA5 but not sF11R correlated negatively with VRI outcome (r = −0.439, *p* = 0.028; Table 2). Furthermore, the correlation between sF11R and ANXA5 was lost in this group of patients (Table 2). In the group of patients with HbA1c > 6.5%, sF11R levels correlated negatively with VRI outcome and total NO levels (r = −0.240, *p* = 0.018 and r = −0.363, *p* = 0.0001, respectively; Table 2) and correlated positively with ANXA2 levels (r = 0.282, *p* = 0.005; Table 2). In addition, ANXA5 levels correlated negatively with CIMT outcome and total NO (r = −0.225, *p* = 0.026; r = −0.412, *p* = 0.0001, respectively; Table 2) in this group of patients.

### 3.3. Linear Regression Analysis between Plasma sF11R, ANXA5, and Vascular Outcomes

Univariable regression analysis revealed that sF11R was significantly associated with ANXA5 in the total population and in the group with HbA1c > 6.5% (β = 0.250, *p* = 0.005 and β = 0.276, *p* = 0.006, respectively; Table 3). In contrast, there was no significant association between sF11R levels and indices of vascular function PWV, VRI, and CIMT (Table 3).

### 3.4. Multiple Linear Regression Analyses between Circulating sF11R and ANXA5

Multivariate regression analysis showed that plasma sF11R levels were independently associated with ANXA5 in the total population (model III: β = −0.366, *p* = 0.007; Table 4) and in poorly-controlled patients with HbA1c > 6.5% (model III: β = −0.425, *p* = 0.008; Table 4), but not in well-controlled patients with HbA1c ≤ 6.5%, after adjusting the model for multiple independent variables, such as gender, age, weight, hypertension, dyslipidemia, stroke, smoking, creatinine, total cholesterol, LDLc, HDLc, triglycerides, diabetes duration, and total NO (Table 4).

### 3.5. Multiple Linear Regression Analyses According to Quartiles of Plasma sF11R and ANXA5

To further assess the association between circulating sF11R and ANXA5, we categorized the total population into quartiles of circulating sF11R and ANXA5 (lower, interquartile, and higher quartile; Table 5 and Table 6). The association between sF11R and ANXA5 was highly significant within the lowest quartile (Q1, <115.92 pg/mL, Model III; *p* < 0.034) and in the highest quartile of sF11R (Q4, >199.02 pg/mL, Model I; *p* < 0.42); Table 5. In contrast, the association between sF11R and ANXA5 was significant among participants in the highest quartile of ANXA5 (Q4, >0.385 ng/mL, Model I; *p* < 0.004; Table 6). The association between sF11R and ANXA5 was attenuated among participants in the highest quartiles of both sF11R and ANXA5 in the adjusted Model 3 (Table 5 and Table 6, respectively).

### 3.6. Effects of Medication Use on the Association between sF11R and ANXA5

Multiple regression analysis demonstrated that the use of various medication by participants did not result in any significant effect on the association between plasma sF11R and ANXA5 levels in the total population or in the poorly-controlled patients (Table 7).

## 4. Discussion

The present study aimed to test the hypothesis that abnormal levels of circulating sF11R and ANXA5 could influence endothelial function outcome in patients with T2DM. There is no or very little information about the association between plasma sF11R and ANXA5 and vascular dysfunction in T2DM. To our knowledge this is the first study characterizing the association between sF11R and ANXA5 levels in T2DM. Although much attention has been focused on the atherothrombotic state in diabetes, our study clearly documents a positive association between circulating sF11R and ANXA5 in poorly-controlled diabetic patients, but not in well-controlled patients. Additionally, sF11R and ANXA5 levels were not associated with vascular endothelial function indices, suggesting that sF11R and ANXA5 may not be reliable markers of endothelial dysfunction and subclinical atherosclerosis in diabetes. F11R has been detected in circulating plasma at the range of pg/mL due to shedding from endothelial cells and platelets by proteases action and/or other mechanisms, resulting in the release of the extracellular domain of F11R into the circulation as sF11R [16,28]. We have previously demonstrated a positive correlation between elevated levels of circulating sF11R and factors of inflammation in hemodialysis patients from a predominantly African American cohort [18]. Furthermore, similar studies reported increased levels of sF11R in hypertensive and CAD patients [19,29]. In this study, we found that levels of circulating sF11R were notably higher than previously reported values for hemodialysis, hypertensive, and patients with normal or nonobstructive disease, suggesting a role of sF11R in the pathophysiology of diabetes [18,19,29]. While, several studies reported abnormal elevations of circulating ANXA5 in familial hypercholesterolemia, hypertensive patients, and patients with myocardial infarction [30,31,32], levels of ANXA5 among the patients of current study were within normal range (0–2 ng/mL), similar to those reported in healthy population [24,33]. Interestingly, we found that sF11R levels correlated positively with ANXA5 levels. In addition, our study showed that both sF11R and ANXA5 levels correlated negatively with VRI outcome and total NO.

It is well established that uncontrolled glycaemia leads to impairment of NO production, which may result in accelerated vascular complications in diabetic patients. Studies have shown that NO production inhibits platelet activation, aggregation, and adhesion to the endothelium, preventing further platelet recruitment from causing pathological thrombosis [34,35,36]. Studies proposed that changes in NO bioavailability were attributed to impairment of nitric oxide synthase (NOS) activity due to chronic glycemia, consequently leading to accelerated diabetic complications and comorbidities. Some studies reported increased NO levels in diabetic patients [37,38], whereas others reported reduced levels of NO [9,39,40]. Although total NO levels in poorly-controlled patients were not significantly different from levels in well-controlled patients, our study revealed that both sF11R and ANXA5 levels correlated inversely with total NO in poorly-controlled patients, but not in well-controlled patients. This finding is consistent with studies reporting the impact of NO on platelet dysfunction in diabetes [1,41,42,43].

Diabetic macrovascular complications are strongly interconnected with microvascular diseases promoting atherosclerosis development. The sequence of apparition of these vascular complications is still unclear; furthermore, it is uncertain if the two complications progress simultaneously or independently. In the current study, we found that none of the circulating sF11R and ANXA5 could independently predictvascular function, which suggests that plasma levels of sF11R and ANXA5 may not be considered reliable indicators for the development and progression of vascular complications in T2DM. It is noteworthy that the lack of associations between sF11R and ANXA5 with vascular outcome could be due to single basal determination of these two circulating markers, and it remains unclear whether the results would differ substantially with repeated measurements during diabetes. A recent study showed that mRNA and protein levels of sF11R were increased in the atherosclerotic plaques of patients with advanced aortic and peripheral vascular disease [44]. Our finding of a lack of association between sF11R and vascular indices is in disagreement with a previous study reporting that plasma levels of sF11R were independently associated with the presence and severity of CAD [29]. One plausible explanation of this discrepancy could relate to differences in the study population involving non-diabetic patients with angiographically defined CAD [29]. With regards to circulating ANXA5, studies have also reported a great abundance of ANXA5 in advanced atheroma; nevertheless, while ANXA5 level is known for its antithrombotic role in the formation of arterial thrombosis, it might also contribute to plaque volume increase during disease progression [45]. The relationship of endogenous ANXA5 with atherosclerotic complications is not well defined. While the levels of circulating ANXA5 have been shown to be associated with the severity of coronary stenosis [46] and subclinical atherosclerosis outcomes in patients with T2DM or systemic lupus erythematosus [33,47], another study revealed no direct association between ANXA5 levels and CIMT progression [30], consistent with the present study. The differences between all the above reported studies could be related to study design, population sample size, and/or the nature and progression of disease among patients. In fact, a recent study demonstrated that poor glycemic control in diabetic patients could trigger, under certain conditions, a shift toward pro-thrombotic and anti-fibrinolytic states [48]. It is conceivable that the expression of ANXA5 on the cell surface is reduced due to inefficient shielding of negatively charged phospholipids from the blood; however, it is unclear whether the resulting endogenous pool of circulating ANXA5 could directly influence the development of atherosclerotic lesions or simply that changes in plasma levels during disease progression may be a consequence of the extent of vascular complications. We found that sF11R and ANXA5 correlated inversely with VRI and CIMT, respectively. However, none of these two parameters was independently associated with endothelial dysfunction and subclinical atherosclerosis, suggesting that circulating sF11R and ANXA5 might have a minor impact on the pathophysiology of atherosclerosis in diabetic patients. The present study cannot elucidate whether circulating levels of sF11R and ANXA5 are causally involved in the development of microvascular and macrovascular diabetic complications, or whether the observed sF11R/ANXA5 association reflects the extent of diabetes, which may not necessarily translate to poor vascular outcome. Nevertheless, the positive association between sF11R and ANXA5 in poorly-controlled diabetic patients should be examined in order to understand its true physiological impact.

sF11R is mainly expressed in epithelial and endothelial cell tight junctions, and also expressed on circulating platelets and leukocytes. In contrast, given the abundant levels of ANXA5 in all cells and tissues, except neurons, one possible mechanism behind the significant correlation between sF11r and ANXA5 in the poorly-controlled diabetic patients might potentially be associated with the release of these proteins by a pool of extracellular vesicles that could originat from the same source of cellular compartments, such as platelets and endothelial cells. The identification of the cellular origins of sF11R and ANXA5 and the determination of the impact of thrombotic and inflammatory factors on sF11R/ANXA5 association in T2DM warrant further investigation.

Several limitations of the current study must be considered. First, this is a retrospective cross-sectional study, with a relatively small sample size and from a single center, and this may have limited the power to detect weak correlations among the study groups; however, the sample size was sufficient to demonstrate strong positive association between sF11R and ANXA5 levels among the groups. Second, we did not exclude patients with prior anti-thrombotic/inti-inflammatory drug use, which may have biased the prognostic value of measured parameters, including indices of vascular function. Lastly, the study population represents a relatively heterogeneous, multi-ethnic community-based T2DM cohort, which may have contributed to individual intra-variability measurements. We are aware of differences in race that could account for discrepancies among studies; therefore, this may limit our ability to extend this investigation to a more rigorous analysis of the role of sF11R/ANXA5 in diabetes.

In summary, it is conceivable that the complexity of diabetes physiopathology might directly or indirectly result in interactions between circulating sF11R and ANXA5 and other diabetes-risk factors, such inflammation, which requires further investigation. To elucidate the pathophysiological role of sF11R and ANXA5 in T2DM, further evidence, especially from longitudinal studies including other racial groups, is required.

## Figures and Tables

**Table 1:** Baseline characteristics of the total study population and stratified groups based on HbA1c levels.

Baseline Characteristics	Total Population (N = 125)	Well-Controlled Patients (HbA1c ≤ 6.5%) (N = 27)	Poorly-Controlled Patients (HbA1c > 6.5%) (N = 98)	*p*-Value
**Age (years)** * Mean (SD)				
	59.68.1 (7.80)	59.10 (9.50)	59.84 (7.31)	0.347
**Weight (kg)** Median (IQR)				
	83.10 (72.64–98.01)	80.81 (74.46–92.62)	83.32 (72.64–103.74)	0.269
**Height (cm)** Median (IQR)				
	165.10 (160.01–172.72)	165.10 (160.00–172.72)	165.10 (160.02–172.72)	0.493
**Waist Circumference (cm)** Mean (SD)				
	99.00 (0, 152)	92.00 (86.36–102.00)	101.60 (90.25–109.22)	0.011
**BMI (kg/m^2^)** Median (IQR)				
	29.86 (26.74–35.01)	30.84 (27.32–32.65)	29.71 (26.14–36.53)	0.237
**Systolic BP (mmHg)** Median (IQR)				
	130.00 (120.00–146.00)	126.00 (115.00–132.00)	131.50 (121.00–148.00)	0.760
Diastolic BP (mmHg) * Mean (SD)				
	75.69 (10.70)	74.63 (9.54)	75.98 (11.03)	0.018
**HbA1c (%)** Median (IQR)				
	8.06 (6.70–9.30)	6.20 (5.80–6.30)	8.00 (7.00–10.00)	0.0001
**Diabetes duration (year)** Median (IQR)				
	10.00 (4.25–14.75)	6.00 (4.00–10.00)	10.00 (5.00–15.75)	0.001
**Total Cholesterol (mg/dL)** Median (IQR)				
	169.00 (148.00–194.50)	182.50 (156.00–202.75)	167.00 (145.00–189.00)	0.211
**LDL-c (mg/dL)** Median (IQR)				
	90.50 (70.60–109.60)	94.55 (80.65–123.38)	90.50 (68.10–107.60)	0.427
HDL-c (mg/dL) * Mean (SD)				
	55.51 (17.80)	58.90 (19.95)	54.58 (17.17)	0.510
**Triglycerides (mg/dL)** Median (IQR)				
	98.00 (73.50–128.50)	91.00 (71.00–127.00)	101.00 (73.75–129.25)	0.510
**ASCVD Score (%)** Median (IQR)				
	19.25 (11.23–29.08)	17.20 (7.00, 33.00)	19.50 (12.00–28.60)	0.295
**PWV (m/s)** Median (IQR)				
	8.10 (6.55–10.20)	7.90 (6.30–10.10)	8.15 (6.80–10.20)	0.943
VRI * Mean (SD)				
	1.16 (0.50)	1.17 (0.40)	1.15 (0.52)	0.778
**CIMT (mm)** Median (IQR)				
	0.65 (0.56–0.73)	0.67 (0.57–0.82)	0.64 (0.56–0.72)	0.657
**Platelet Count (×10^3^/mL)** Median (IQR)				
	241.50 (199.00–297.75)	237.00 (196.00–290.00)	246.00 (201.00–302.00)	0.320
**Creatinine (mg/dL)** Median (IQR)				
	0.89 (0.77–1.22)	0.91 (0.82–1.23)	0.89 (0.76–1.21)	0.217
**Total Nitric oxide (µmol/L)** Median (IQR)				
	20.09 (16.27–30.74)	20.09 (16.37–27.78)	20.03 (16.13–32.83)	0.459
**F11R/JAM-A (pg/mL)** Median (IQR)				
	158.8 (115.92–199.02)	153.16 (107.25–239.74)	159.62 (116.90–194.43)	0.829
**ANXA5 (ng/mL)** Median (IQR)				
	0.22 (0.07–0.39)	0.32 (0.07–0.43)	0.21 (0.07–0.33)	0.597
**Insulin,** n (%)	35 (28.0)	6 (22.2)	19 (34.3)	0.899
**Sulfonylurea,** n (%)	25 (20.0)	3 (11.1)	16 (28.4)	0.331
**Metformin,** n (%)	91 (72.8)	22 (81.5)	37 (65.7)	0.900
**DPP-4 inhibitors,** n (%)	41 (32.8)	6 (22.2)	1 (1.8)	0.722
**GLP-1 agonists,** n (%)	1 (0.8)	4 (14.8)	8 (14.9)	0.193
**SGLT2 inhibitors,** n (%)	16 (12.8)	4 (14.8)	1 (1.5)	0.997
**Thiazolidinediones,** n (%)	16 (12.8)	4 (14.8)	8 (14.9)	0.999
**Alpha glucosidase inhibitors,** n (%)	1 (0.8)	4 (14.8)	1 (1.5)	0.777
**Calcium channel blockers,** n (%)	38 (30.4)	7 (25.9)	14 (23.9)	0.008
**ACE inhibitors,** n (%)	43 (34.4)	5 (18.5)	19 (34.3)	0.900
**Beta blockers,** n (%)	27 (21.6)	4 (14.8)	17 (29.9)	0.067
**Alpha2 agonists,** n (%)	2 (1.6)	4 (14.8)	15 (26.9)	0.658
**Nitrates,** n (%)	6 (4.8)	4 (14.8)	5 (9.0)	0.297
**Anti-platelets,** n (%)	37 (29.6)	7 (25.9)	15 (26.9)	0.688
**Statins,** n (%)	71 (56.8)	15 (55.6)	35 (61.2)	0.219

Data are presented for continuous variables as mean (standard deviation, SD) or median (interquartile range, IQR), and as frequencies (percentages) for categorical variables. *, data are normally distributed. BMI, body mass index; BP, blood pressure; HbA1c, hemoglobin A1c; LDLc, low density lipoprotein cholesterol; HDLc, high density lipoprotein cholesterol; ASCVD, atherosclerotic cardiovascular disease; PWV, pulse waive velocity; VRI, vascular reactivity index; CIMT, carotid intima-media thickness; F11R/JAM-A, junctional adhesion molecule A; ANXA5, annexin A5; DDP4, dipeptidyl peptidase 4; GLP1, glucagon-like peptide-1; SGLT2, sodium glucose transport protein 2; ACE, angiotensin-converting enzyme.

**Table 2:** Correlation between plasma sF11R and ANXA5 levels with vascular outcome and lipid parameters in the total population and stratified groups based on HbA1c levels.

	PWV	VRI	CIMT	TC	LDL-c	HDL-c	TG	Total NO	ANXA5	sF11R
**TOTAL POPULATION (N = 125)**										
**sF11R**	0.050 (0.584)	−0.201 ^a^ (0.024)	−0.049 (0.588)	−0.010 (0.911)	0.075 (0.413)	−0.133 (0.145)	0.095 (0.300)	−0.357 ^b^ (0.0001)	0.250 ^a^ (0.005)	1.000 .
**ANXA5**	0.103 (0.264)	−0.179 ^a^ (0.049)	−0.136 (0.131)	−0.036 (0.693)	0.052 (0.570)	−0.083 (0.364)	−0.008 (0.929)	−0.351 ^b^ (0.0001)	1.000 .	0.250 ^a^ (0.005)
**WELL-CONTROLLED PATIENTS, HbA1c ≤ 6.5 % (N = 27)**										
**sF11R**	0.068 (0.753)	−0.082 (0.697)	−0.018 (0.930)	0.159 (0.437)	0.219 (0.282)	−0.014 (0.944)	0.093 (0.646)	−0.327 (0.096)	−0.043 (0.831)	1.000 .
**ANXA5**	0.306 (0.146)	−0.439 ^a^ (0.028)	0.080 (0.692)	−0.105 (0.610)	−0.115 (0.575)	−0.105 (0.608)	0.089 (0.660)	−0.053 (0.792)	1.000 .	−0.043 (0.831)
**POORLY-CONTROLLED PATIENTS, HbA1c > 6.5 % (N = 98)**										
**sF11R**	0.061 (0.556)	−0.240 ^a^ (0.018)	−0.076 (0.455)	−0.053 (0.607)	0.043 (0.679)	−0.182 (0.077)	0.099 (0.343)	−0.363 ^b^ (0.0001)	0.282 ^a^ (0.005)	1.000 .
**ANXA5**	0.075 (0.471)	−0.143 (0.161)	−0.225 ^a^ (0.026))	−0.043 (0.677)	0.089 (0.391)	−0.107 (0.304)	−0.043 (0.681)	−0.412 ^b^ (0.0001)	1.000	0.282 ^a^ (0.005)

Results are expressed as R, Spearman’s rho coefficient, and (p-value) for 2-tailed significance: ^a^, *p* < 0.05; ^b^, *p* < 0.0001. PWV: pulse wave velocity; VRI: vascular reactivity index; wave; CIMT, carotid intima-media thickness; TC: total cholesterol; LDL-c: low-density lipoprotein cholesterol; HDL-c: high-density lipoprotein cholesterol; TG: triglycerides; NO: nitric oxide.

**Table 3:** Univariate analysis of association between plasma sF11R, ANXA5 levels, and vascular outcome.

	Total Population			Patients with HbA1c ≤ 6.5 %			Patients with HbA1c > 6.5 %		
	Standardized β Coefficient	95% CI (Min) (Max)	*p*-Value	Standardized β Coefficient	95% CI (Min) (Max)	*p*-Value	Standardized β Coefficient	95% CI (Min) (Max)	*p*-Value
**sF11R ^a^**	0.250	0.0001 0.001	0.005	−0.027	−0.002 0.001	0.895	0.276	0.0001 0.001	0.006
**sF11R ^b^**	−0.061	−0.004 0.002	0.505	0.046	−0.012 0.015	0.833	−0.075	−0.005 0.002	0.468
**sF11R ^c^**	−0.076	−0.001 0.0001	0.407	0.037	−0.002 0.002	0.860	−0.086	−0.001 0.0001	0.402
**sF11R ^d^**	−0.098	0.0001 0.0001	0.275	−0.049	−0.001 0.001	0.810	−0.105	0.0001 0.001	0.302
**sF11R ^e^**	−0.090	−0.064 0.021	0.316	−0.204	−0.071 0.023	0.308	−0.092	−0.071 0.027	0.370

Dependent variables: a: ANXA5; b: PWV; c: VRI; d: CIMT; e: NO. Data are expressed as standardized regression coefficient β and 95% CI (confidence intervals), with lower and upper bound values (min and max). PWV, pulse wave velocity; VRI, vascular reactivity index; CIMT, carotid intima-media thickness; F11R/JAM-A, junctional adhesion molecule A; ANXA5, annexin A5; NO, nitric oxide.

**Table 4:** Multiple regression analysis of the association between plasma sF11R and ANXA5 levels.

Variables	Total Population (N = 125)			Well-Controlled Patients HbA1c ≤ 6.5% (N = 27)			Poorly Controlled Patients HbA1c > 6.5% (N = 98)		
	Model I (R Square = 0.084)			Model I (R Square = 0.221)			Model I (R Square = 0.106)		
**MODEL I**	**β**	**95% CI** **(Min)** **(Max)**	***p*-value**	**β**	**95% CI** **(Min)** **(Max)**	***p*-value**	**β**	**95% CI** **(Min)** **(Max)**	***p*-value**
**sF11R**	0.245	0.0001 0.001	0.006	−0.159	−0.002 0.001	0.432	0.266	0.0001 0.001	0.008
**Sex**	−0.044	−0.161 0.097	0.624	−0.466	−0.471 0.024	0.031	0.027	−0.134 0.175	0.790
**Age**	−0.018	−0.009 0.007	0.846	0.148	−0.008 0.016	0.473	−0.034	−0.012 0.009	0.739
**Weight**	−0.137	−0.005 0.00	0.137	0.192	−0.004 0.012	0.342	−0.182	−0.007 0.0001	0.086
	**Model II (R Square = 0.143)**			**Model II (R Square = 0.445)**			**Model II (R Square = 0.198)**		
**MODEL II**	**β**	**95% CI** **(Min)** **(Max)**	***p*-value**	**β**	**95% CI** **(Min)** **(Max)**	***p*-value**	**β**	**95% CI** **(Min)** **(Max)**	***p*-value**
**sF11R**	0.308	0.0001 0.001	0.003	−0.239	−0.033 0.001	0.327	0.355	0.0001 0.001	0.002
**Sex**	0.050	−0.129 0.204	0.654	−0.252	−0.479 0.205	0.399	0.134	−0.091 0.305	0.286
**Age**	−0.042	−0.013 0.008	0.691	0.348	−0.009 0.032	0.238	−0.025	−0.014 0.012	0.833
**Weight**	−0.091	−0.006 0.002	0.400	0.714	0.001 0.032	0.041	−0.146	−0.007 0.002	0.230
**Hypertension**	0.151	−0.057 0.373	0.147	0.027	−0.334 0.370	0.912	0.134	−0.114 0.425	0.254
**Stroke**	−0.061	−0.075 0.040	0.551	−0.182	−0.927 0.467	0.486	−0.071	−0.081 0.042	0.538
**Dyslipidemia**	0.112	−0.088 0.300	0.280	−0.037	−0.343 0.298	0.882	0.151	−0.084 0.408	0.193
**Smoking**	−0.098	−0.268 0.106	0.391	−0.232	−0.532 0.256	0.460	−0.118	−0.325 0.116	0.347
	**Model III** **(R Square = 0.237)**			**Model III** **(R Square = 1.000)**			**Model III** **(R Square = 0.318)**		
**MODEL III**	**β**	**95% CI** **(Min)** **(Max)**	***p*-value**	**β**	**95% CI** **(Min)** **(Max)**	***p*-value**	**β**	**95% CI** **(Min)** **(Max)**	***p*-value**
**sF11R**	0.366	0.0001 0.001	0.007	0.247	0.001 0.001	NS	0.425	0.0001 0.001	0.008
**Sex**	−0.001	−0.235 0.233	0.993	−1.002	−0.604 −0.604	NS	0.165	−0.161 0.391	0.406
**Age**	0.096	−0.008 0.017	0.496	1.077	0.041 0.041	NS	0.129	−0.010 0.022	0.438
**Weight**	0.024	−0.004 0.005	0.858	−0.190	−0.005 −0.005	NS	−0.022	−0.005 0.005	0.890
**Hypertension**	0.177	−0.085 0.409	0.193	−1.094	0.839 0.839	NS	0.108	−0.212 0.420	0.510
**Stroke**	−0.045	−0.066 0.046	0.720	−0.504	−0.622 −0.622	NS	−0.045	−0.068 0.050	0.759
**Dyslipidemia**	0.165	−0.098 0.366	0.251	−0.373	−0.258 −0.258	NS	0.213	−0.116 0.477	0.227
**Smoking**	−0.088	−0.293 0.160	0.558	0.963	0.622 0.622	NS	−0.138	−0.373 0.153	0.402
**Creatinine**	−0.030	−0.238 0.195	0.842	−0.412	−0.346 −0.346	NS	−0.078	−0.293 0.184	0.645
**Total** **Cholesterol**	−0.360	−0.019 0.013	0.690	−34.580	−0.376 −0.376	NS	0.075	-0.017 0.018	0.939
**LDL-c**	0.267	−0.014 0.020	0.735	30.775	0.369 0.369	NS	−0.090	−0.019 0.017	0.918
**HDL-c**	0.097	−0.016 0.020	0.830	15.155	0.403 0.403	NS	−0.097	−0.022 0.018	0.851
**Triglycerides**	0.058	−0.004 0.005	0.836	11.990	0.075 0.075	NS	−0.106	−0.006 0.004	0.728
**Diabetes** **Duration**	−0.172	−0.018 0.004	0.219	−0.572	−0.034 −0.034	NS	−0.160	−0.018 0.006	0.302
**Total NO**	−0.081	−0.002 0.001	0.524	0.952	0.034 0.034	NS	−0.069	−0.002 0.002	0.643

Dependent variable: ANXA5; R Square: proportion of variance between variables in linear regression model. Linear regression was performed using 3 separates models (I, II, III) with various variables as discussed in the Section 3. Data are expressed as standardized regression coefficient β and 95% CI (confidence intervals), with lower and upper bound values (min and max). LDL-c, low density lipoprotein cholesterol; HDLc, high density lipoprotein cholesterol; PWV, pulse wave velocity; VRI, vascular reactivity index; CIMT, carotid intima-media thickness; F11R/JAM-A, junctional adhesion molecule A; ANXA5, annexin A5; NO, nitric oxide.

**Table 5:** Association between sF11R and ANXA5 according to quartiles of circulating plasma sF11R levels.

	sF11R Quartiles (pg/mL)											
	Q1 (<115.92) N = 30			Q2 (115.92–158.72) N = 31			Q3 (158.72–199.02) N = 30			Q4 (>199.02) N = 31		
	Standardized β Coefficient	95% CI (Min) (Max)	*p*-Value	Standardized β Coefficient	95% CI (Min) (Max)	*p*-Value	Standardized β Coefficient	95% CI (Min) (Max)	*p*-Value	Standardized β Coefficient	95% CI (Min) (Max)	*p*-Value
**Model I**	0.008	−0.008 0.008	0.968	0.169	−0.004 0.011	0.169	−0.048	−0.017 0.014	0.819	0.361	0.0001 0.001	0.064
**Model II**	0.304	−0.006 0.019	0.263	0.198	−0.008 0.016	0.492	−0.145	−0.027 0.017	0.615	0.539	0.0001 0.002	0.042
**Model III**	2.214	0.006 0.080	0.034	0.464	−0.034 0.051	0.475	−0.764	−0.076 0.041	0.415	0.664	−0.015 0.018	0.782

Dependent variable: ANXA5. Model I: adjusted for sex, age, and weight. Model II: adjusted for sex, age, weight, hypertension, stroke, dyslipidemia, and smoking. Model III: adjusted for sex, age, weight, hypertension, stroke, dyslipidemia, smoking, creatinine, total cholesterol, LDL-c, IDL-c, HDL-c, triglycerides, duration of diabetes, and total nitric oxide. Data are expressed as standardized regression coefficient β and 95% CI (confidence intervals), with lower and upper bound values (min and max).

**Table 6:** Association between sF11R and ANXA5 according to quartiles of circulating plasma ANXA5 levels.

	ANXA5 Quartiles (ng/mL)											
	Q1 (<0.070) N = 28			Q2 (0.070–0.220) N = 29			Q3 (0.220–0.385) N = 31			Q4 (>0.385) N = 31		
	β Coefficient	95% CI (Min) (Max)	*p*-Value	β Coefficient	95% CI (Min) (Max)	*p*-Value	β Coefficient	95% CI (Min) (Max)	*p*-Value	β Coefficient	95% CI (Min) (Max)	*p*-Value
**Model** **I**	0.014	−2369.6 2539.0	0.944	−0.146	−679.3 323.4	0.471	0.320	−334.0 3989.3	0.094	0.520	67.22 310.79	0.004
**Model II**	−0.106	−3436.5 2197.8	0.647	0.112	−727.6 995.7	0.740	0.080	−1541.7 2307.8	0.677	0.496	−8.32 376.59	0.059
**Model III**	−0.211	−3937.3 1467.2	0.328	−1.324	−1333.5 0.051	0.999	−0.229	−1297.2 −1297.2	0.999	1.085	536.54 536.54	0.999

Dependent variable: sF11R. Model I: adjusted for sex, age, and weight. Model II: adjusted for sex, age, weight, hypertension, stroke, dyslipidemia, and smoking. Model III: adjusted for sex, age, weight, hypertension, stroke, dyslipidemia, smoking, creatinine, total cholesterol, LDL-c, IDL-c, HDL-c, triglycerides, duration of diabetes, and total nitric oxide. Data are expressed as standardized regression coefficient β and 95% CI (confidence intervals), with lower and upper bound values (min and max).

**Table 7:** Influence of medication on the association between circulating sF11R and ANXA5 levels.

Variables	Total Population (N = 125)			Well-Controlled Patients HbA1c ≤ 6.5% (N = 27)			Poorly Controlled Patients HbA1c > 6.5% (N = 98)		
	R Square = 0.160			R Square = 0.664			R Square = 0.229		
	β	95% CI (Min) (Max)	*p*-Value	β	95% CI (Min) (Max)	*p*-Value	β	95% CI (Min) (Max)	*p*-Value
**sF11R**	0.373	0.0001 0.001	0.001	−0.024	−0.002 0.001	0.902	0.450	0.0001 0.001	0.001
**Insulin**	−0.094	−0.205 0.073	0.348	−0.084	−0.296 0.395	0.761	−0.095	−0.229 0.090	0.387
**Sulfonylurea**	−0.010	−0.164 0.147	0.917	−0.069	−0.433 0.328	0.767	0.047	−0.137 0.212	0.670
**Metformin**	0.024	−0.177 0.219	0.833	0.252	−0.339 0.975	0.313	0.030	−0.186 0.239	0.805
**DPP4**	−0.019	−0.149 0.123	0.850	−0.228	−0.422 0.154	0.332	−0.056	−0.195 0.116	0.613
**GLP-1 agonists**	−0.031	−0.767 0.555	0.751	Nd	Nd	Nd	−0.031	−0.790 0.588	0.771
**Alpha-glucosidase inhibitors**	−0.038	−0.810 0.547	0.701	Nd	Nd	Nd	−0.048	−0.861 0.551	0.664
**Calcium Channel blockers**	−0.035	−0.164 0.116	0.736	−0.122	−0.314 0.178	0.558	0.030	−0.141 0.185	0.791
**ACE inhibitors**	−0.021	−0.150 0.121	0.833	−0.238	−0.490 0.193	0.361	0.61	−0.116 0.201	0.594
**Beta Blockers**	−0.091	−0.253 0.114	0.452	0.731	0.137 0.855	0.011	−0.251	−0.408 0.015	0.068
**Alpha-2 agonists**	−0.119	−0.875 0.290	0.321	Nd	Nd	Nd	−0.145	−0.951 0.285	0.286
**Nitrates**	0.144	−0.103 0.519	0.188	Nd	Nd	Nd	0.212	−0.041 0.617	0.085
**Anti-platelets**	0.011	−0.135 0.150	0.918	−0.223	−0.378 0.128	0.303	0.075	−0.112 0.221	0.516
**Statins**	0.169	−0.033 0.266	0.124	0.202	−0.130 0.349	0.340	0.118	−0.095 0.267	0.346

ANXA5 was used as dependent variable; R Square: proportion of variance between variables in linear regression model. Data are expressed as standardized regression coefficient β and 95% CI (confidence intervals), with lower and upper bound values (min and max). Nd: variables not computed due to missing correlations or values were constant. Variables such as thiazolidinediones and SGTL2 inhibitors were not included in model analysis due to missing correlations. DDP4, dipeptidyl peptidase 4; GLP1, glucagon-like peptide-1; SGLT2, sodium glucose transport protein 2; ACE, angiotensin-converting enzyme.

## Data Availability

Not applicable.
